# Healthcare Workers’ Safety; A Necessity for a Robust Health System

**DOI:** 10.5334/aogh.4167

**Published:** 2023-09-06

**Authors:** Kengo Nathan Ezie, Felix Amekpor, Godfred Yawson Scott, Angyiba Serge Andigema, Shuaibu Saidu Musa, Berjo Dongmo Takoutsing, Don Eliseo Lucero-Prisno III

**Affiliations:** 1 Faculty of Medicine and Biomedical Sciences, University of Garoua, Garoua, CM; 2 Research Division, Winners Foundation, Yaounde, CM; 3 Department of Medical Diagnostics Institution, Kwame Nkrumah University of Science and Technology, Ghana; 4 Department of Medical Diagnostics, Kwame Nkrumah University of Science and Technology, Ghana; 5 Department of Microbiology, Immunology and Hematology, University of Dschang, CM; 6 Department of Nursing Science, Ahmadu Bello University, Zaria, NG; 7 Research Department, Association of Future African Neurosurgeons, Yaounde, CM; 8 Department of Global Health and Development, London School of hygiene and Tropical Medicine, London, UK

**Keywords:** healthcare workers, occupational medicine, patient’s well-being

## Abstract

It is a prevalent misconception that healthcare professionals are specialists and thus can effectively manage their health. This is probably true, but given recent pandemics and the rise in violence in medical settings, one is compelled to question whether their health and safety are sufficient for a robust healthcare system. This is important because protecting and promoting the health, safety, and well-being of health workers will improve the quality of patient care and increase the resilience of health services in the face of outbreaks and public health emergencies. We thus strive to answer this question and suggest potential remedies to this growing public health issue.

The healthcare industry comprises of people engaged in health-related activities as defined by the International Standard Classification of Occupations (ISCO-08) [1]. According to ISCO-08, both medical and paramedical personnels fall under this category. It is a field associated with multiple risks given that employees are frequently exposed to a wide range of health and safety hazards while performing their duties [1]. The related risks include: exposures to radiation, chemicals, biological pathogens; physical and psychosocial drawbacks; and a rise in suicide rates among medical workers [1,2]. There is therefore a need to identify and address these problems in order to ensure a robust healthcare system and achieve shared health goals.

Exposure to harmful chemicals such as ethylene oxide and glutaraldehyde are detrimental to the health of healthcare workers (HCWs) [2]. Similarly, contact with biological pathogens including Coronavirus disease 2019 (COVID-19), *Mycobacterium tuberculosis*, Hepatitis B and C viruses, and the human immunodeficiency virus (HIV) are just some of the potential biological hazards that HCWs face [2]. A reports from the World Health Organisation (WHO) on November 7, 2022, states that latent tuberculosis affects 54% of healthcare personnel in lower-and middle-income countries (LMICs), which is 25 times greater than the general population [1]. Unsatisfactory hand hygiene before and after interaction with patients, insufficient personal protective equipment (PPE), direct patient interaction, and long daily contact hours are all linked to HCWs’ risk associated with these exposures [3].

Regarding psychosocial well-being, a non-conducive working environment, high levels of stress and long working hours make it difficult for healthcare practitioners to adopt healthy living practices [3]. They are also afflicted with disorders like anxiety, panic disorder, sadness, and insomnia. As a result, healthcare professionals face serious health and well-being issues. During the COVID-19 pandemic, 23% of front-line healthcare personnel worldwide reported feeling depressed and anxious, and 39% said they had insomnia [1]. People abusing HCWs is also a growing problem in the global community, and this may hinder the proper management of patient data especially its collection due to fear of abuse. Globally, 63% of HCWs report contact with violence of some type at work [4,5,6].

The physical and professional challenges HCWs face with their job are considerable. In Africa, between 44% to 83% of nurses as opposed to 18% of office employees report experiencing persistent lower back pain as an occupational hazard [1]. During the COVID-19 pandemic, HCWs faced issues with inadequate physical protection, overworked staff, a lack of permanent staff, and floating staff making them more prone to infection [7]. This was coupled with the physical and mental effects of wearing personal protection equipment (PPE), stigma, avoidance, and a lack of recognition [7]. They often have high turnover rates, worker burnout, and low salaries and as a result, many feel underpaid. According to a recent statistic, the healthcare sector has seen a 5% increase in turnover across all of its occupations over the past ten years [8]. Worker dissatisfaction has also been reported and this is a result of poor organization of healthcare infrastructures, daily stress—especially in high-intensity medical operations, and workforce shortages. Dissatisfaction will lead to a drop in the motivation of HCWs, and if this is to be coupled with their job exposure, it renders them vulnerable to inefficiency, reduced job satisfaction, and prone to illnesses and injury. This increases the cost of occupational injury and serves as a burden to the system. Therefore, in the global patient safety action plan 2021–2030, which was endorsed by the 74th World Health Assembly, action on health worker safety was emphasized for patient safety, such as incorporating the protection of the health and safety of healthcare professionals into all aspects of the management of healthcare [9].

Given the implication of HCWs’ health, and as an essential component of the six health system pillars, a disequilibrium is established leading to a decreased healthcare output. Service delivery, information system, infrastructure, finance, and governance are thus affected directly and/indirectly by qualitative and or quantitative health workforce deficiency.

For instance, during pandemics, healthcare institutions are unsure of when HCWs with infections will resume their jobs. This greatly affects service delivery as their colleagues take on long working hours accounting for reported burnout [10,11]. The costs to HCWs’ health in cases of exposure or illness to an extent affects the institutional finances in the form of low output and could slow the implementation of projects such as infrastructural maintenance or expansion. This may impact budgeting by the institutions and cause management difficulties. Moreover, users will avoid structures that are deficient in quantitative or qualitative personnel. The effect of a deficient health workforce on the healthcare system cannot be minimized due to its repercussion on the entire health system.

The health sector bears a hefty financial burden from unsafe working conditions that cause occupational diseases, accidents, and absenteeism (up to 2% of total health spending, according to estimates) [1]. There are currently just 26 national programs and policy structures in place among the 195 WHO Member States for managing the occupational health and safety of health professionals [1]. Globally, improving the safety, well-being, and health of medical personnel contributes to lowering patient harm, which is considered to be responsible for up to 12% of health expenditures in Great Britain in 2017, for instance. Here, the healthcare and social services sector had the greatest annual costs for occupational illnesses and injuries, estimated at the equivalent of US$3.38 billion [1]. It will also raise the effectiveness of essential measures to safeguard the health and safety of healthcare professionals, strengthen the ability of health services to withstand epidemics and other public health emergencies and reduce unnecessary spending on HCWs [12]. “Caring for those who care: guidance for the Creation and Implementation of occupational health and safety programs for health professionals,” a handbook released by the International Labour Organization (ILO) and WHO on February 21, 2022, is intended to improve care for HCWs and would be very necessary for a global healthcare system that is both viable and durable [13].

People engaged in health-related activities face considerable occupational exposures in and out of health crisis periods thus represent a group at risk. HCWs may be tempted to quit if they have reason to believe that staying on the job constitutes a significant and immediate risk to their life or health. To manage the global occupational health and safety of health professionals, the 169 other member nations of the WHO must create national programs and policy structures aimed at protecting HCWs from occupational hazards in line with the recommendations of the 74th World Health Assembly decision. HCW’s equipment should be controlled for quality, psychosocial programs instituted for their well-being and systematic autopsy in case of demise in order to identify possible exposures. Additionally, their motivation should be checked regularly to ensure job satisfaction which is aimed at improving the quality of patient care and building a robust healthcare system.
